# Nanoparticle-insertion scheme to decouple electron injection from laser evolution in laser wakefield acceleration

**DOI:** 10.1038/s41598-022-15125-6

**Published:** 2022-07-01

**Authors:** Jiancai Xu, Leejin Bae, Mohamed Ezzat, Hyung Taek Kim, Jeong Moon Yang, Sang Hwa Lee, Jin Woo Yoon, Jae Hee Sung, Seong Ku Lee, Liangliang Ji, Baifei Shen, Chang Hee Nam

**Affiliations:** 1 State Key Laboratory of High Field Laser Physics, CAS Center for Excellence in Ultra-Intense Laser Science, Shanghai Institute of Optics and Fine Mechanics (SIOM), Chinese Academy of Sciences(CAS), Shanghai, 201800 China; 2 Center for Relativistic Laser Science (CoReLS), Institute for Basic Science, Gwangju, 61005 Republic of Korea; 3 Department of Physics, Chonnam National University, Gwangju, 61186 Republic of Korea; 4 Advanced Photonics Research Institute (APRI), Gwangju Institute of Science and Technology, Gwangju, 61005 Republic of Korea; 5 Department of Physics, Shanghai Normal University, Shanghai, 200234 China; 6 Department of Physics and Photon Science, Gwangju Institute of Science and Technology, Gwangju, 61005 Republic of Korea

**Keywords:** Laser-produced plasmas, Plasma-based accelerators

## Abstract

A localized nanoparticle insertion scheme is developed to decouple electron injection from laser evolution in laser wakefield acceleration. Here we report the experimental realization of a controllable electron injection by the nanoparticle insertion method into a plasma medium, where the injection position is localized within the short range of 100 μm. Nanoparticles were generated by the laser ablation process of a copper blade target using a 3-ns 532-nm laser pulse with fluence above 100 J/cm^2^. The produced electron bunches with a beam charge above 300 pC and divergence of around 12 mrad show the injection probability over 90% after optimizing the ablation laser energy and the temporal delay between the ablation and the main laser pulses. Since this nanoparticle insertion method can avoid the disturbing effects of electron injection process on laser evolution, the stable high-charge injection method can provide a suitable electron injector for multi-GeV electron sources from low-density plasmas.

## Introduction

Laser wakefield acceleration (LWFA) research has made significant progresses in the last 40 years since it promised a high-gradient acceleration field of 100 GV/m in compact configurations^1,2^. The blow-out or bubble regime has been confirmed to be a practical scheme to produce high-energy electron bunches^3^. In the LWFA, an electron injection into a plasma wave is a critical issue to obtain stable and high-quality electron beams. Various electron injection methods have been developed to reproducibly generate high-quality electron bunches with the energy of a few 100’s MeV at high stabilities^4–10^, and these electron sources have been successfully applied in producing x-ray radiations^11–13^. For increasing electron energy further, a low-density plasma over a centimeter-long acceleration length is required to mitigate the dephasing effect in the acceleration process^14^. Several different methods have been proposed to guide the laser pulse over several-centimeter distances in a gas cell or a capillary for multi-GeV electron beam production^15–19^. To date, the maximum electron bunch energy reached 7.8 GeV, which was produced with a plasma density below 10^18^ cm^−3^ based on self-injection and a long acceleration distance of 20 cm^19^. The self-injection method, however, has a limitation of the deformation of laser propagation during the occurrence of highly nonlinear plasma waves.

A suitable injection scheme is essential for generating a stable multi-GeV electron bunch. Since guiding a laser pulse beyond tens of cm is highly desirable during the electron acceleration process, the required injection method should have minor effects on the laser pulse evolution. Current experiments towards multi-GeV electrons are mostly based on self-injection^16,19^ or ionization injection method^15^, which cannot decouple the electron injection process from the laser evolution. A nanoparticle-based electron injection method offers a promising way to solve this problem since the size of nanoparticles is ~ ten’s nanometers and can avoid the modulation of laser pulse evolution from the electron injection process.

The method based on nanometer-sized particles, first proposed in 2007^20^, uses a nanowire to trigger electron injection in plasma. Then nanoparticles are also studied based on multi-dimensional particle-in-cell simulations. These theoretical works showed that high-density ionized electrons from nanowires or nanoparticles induce an intense local electric field, strongly modulate a well-structured plasma wakefield, and thus a large amount of electron charge can be trapped. Moreover, simulations also illustrate that nanoparticle injection in plasma density of 10^17^ cm^−3^ can generate 5 GeV peak electron energy with a 1% relative energy spread^21^, which suggests that a single nanoparticle can trigger electron injection in low-density plasmas. In this paper, a localized nanoparticle insertion method is experimentally demonstrated to induce stable high-charge electron injections. It presents high injection probability over 90% and localized injection position within 100 μm. A 532 nm laser pulse is employed to produce nanoparticles. The fluence of the ablation laser can determine the size and density of nanoparticles, hence the electron beam charge and stability, providing a practical approach to control the beam source as desired. Nanoparticles with size of only a few tens of nanometer cannot cause a noticeable effect on the evolution of the main laser pulse in the plasma medium, but it can induce stable injection with a very high bunch charge. Thus, it decouples the injection process and the laser pulse evolution process, leading to highly collimated stable electron beams with a charge of 100 s’ pC and a large energy spread. This injection method can provide a suitable injector for multi-GeV laser electron accelerators.

## Results

**Figure 1:**
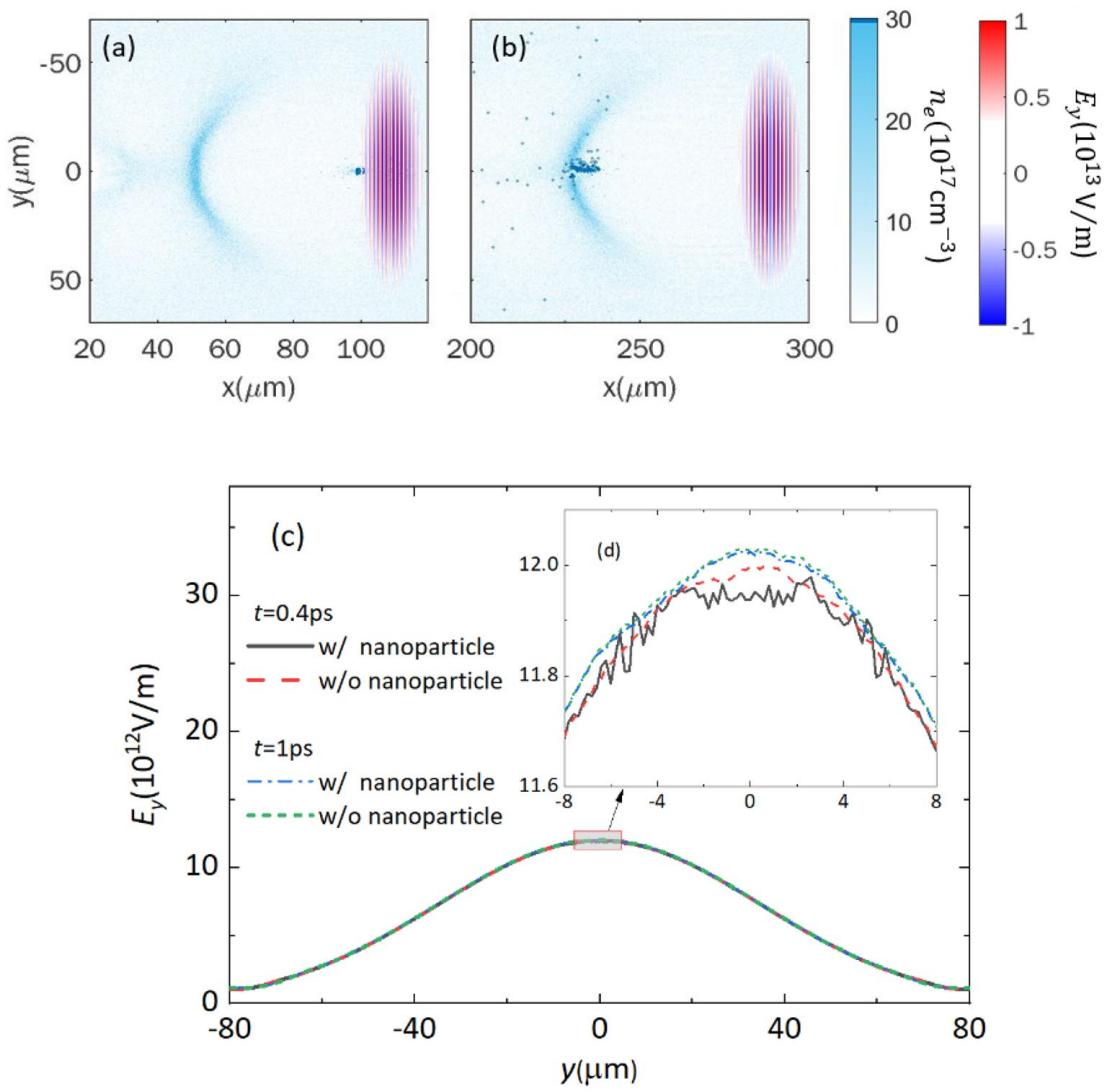
Electron injection induced by a nanoparticle in laser wakefield and evolution of a laser pulse in plasma. (**a**) A nanoparticle induces an intense local field at *x* = 100 μm, as the laser pulse just passes through the nanoparticle at *t* = 0.4 ps, and (**b**) the trapped electrons sit in the backside of the first bubble at *t* = 1 ps. Most electrons from the nanoparticle are trapped into the wakefield. (**c**) Lineout of the laser field *E*_y_ is plotted at different times with/without the nanoparticle, and the central part of *E*_y_ field is zoomed in as the inset figure (**d**).

We investigated the laser pulse evolution process for the nanoparticle-induced electron injection in a low-density plasma with 3D particle-in-cell (PIC) simulations and details of simulation parameters are given in “Methods”. When the nanoparticle is inserted into the plasma medium at *x* = 100 μm, it induces an intense local electric field, modulating the laser-driven plasma wakefield, and thus electrons are injected into the first plasma bubble^21^, as shown in Fig. 1a,b. The beam charge of these trapped electrons is 16 pC in our simulation condition. To see the laser pulse evolution in plasma, snapshots of laser field *E*_y_ lineout along the *y*-axis with/without the nanoparticle are plotted in Fig. 1c. We look into the deformation of the electric field by enlarging the part near the maximum *E*_y_ value along the *x*-axis at *z* = 0, as shown in Fig. 1d. The *E*_y_ lineouts with/without the nanoparticle at 0.4 ps and 1 ps are almost identical, while a slight difference appears at around the peak of the electric field. When the laser pulse passes through the nanoparticle at *t* = 0.4 ps, the electric field *E*_y_, marked as the black solid line, shows a small diffraction pattern by the nanoparticle at around *y* = ±5 μm. The maximum modulation amplitude of the diffraction pattern is about $\text{0.8}\%$ of the maximum *E*_y_ value and gradually decreases during the laser propagation in plasma. The diffraction pattern vanishes at around 60 $\upmu \mathrm{m}$ away from the nanoparticle. When the laser pulse propagates further, no notable difference is seen with/without nanoparticle at *t* = 1 ps, plotted as the blue dot-dash line and green dashed line, respectively. Since the diffraction pattern of the laser pulse induced by a nanoparticle has a small amplitude and a short lifetime, the effect of the 100 nm nanoparticle on the laser evolution in plasma is negligible. The nanoparticles employed in experiments usually have diameters smaller than 100 nm and the nanoparticle-based injection fundamentally avoids the modulation of the laser pulse from the electron injection process. Thus, it decouples the injection process and the laser pulse evolution process, which can be suitable for multi-GeV electron bunch production that requires the guiding of a laser pulse over 10 cm.

Nanoparticles can be generated by laser ablation of a metal material using an intense laser pulse with duration from femtoseconds to nanoseconds. These metal nanoparticles usually have diameters below 100 nm^22,23^. Aluminum nanoparticles, generated by a 3-ns 532-nm ablation laser, have been proposed to trigger electron injection in our previous study^24^. This study demonstrated experimentally that a single nanoparticle is able to trigger electron injection and produce low-divergence electron bunches with a charge of a few pC. In the experimental setup, the aluminum target was set in the bottom of the nozzle and the emitted nanoparticles co-propagated with the gas flow, perpendicular to the main laser pulse, which triggered the electron injection when they met the driving laser pulse. However, in this approach, nanoparticles occupied the whole area of the gas jet, which causes uncertainty in the electron injection position along the laser pulse propagation direction. Lacking in the injection position control limited the generation of stable electron bunches.

**Figure 2:**
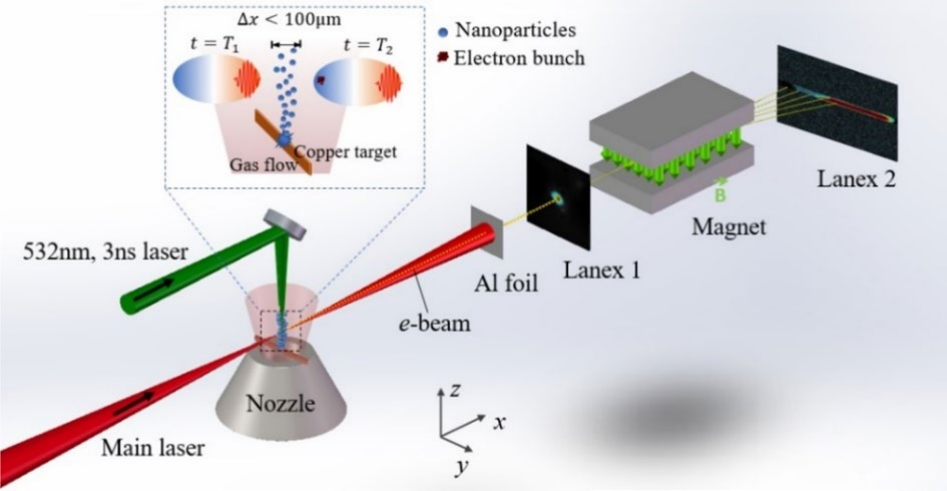
Experimental setup for the nanoparticle-based electron injection in LWFA. The main pulse drives a nonlinear plasma wakefield along the *x*-axis. A copper blade with a thickness of 100 μm is used as an ablation target inserting above the nozzle exit. A 3 ns green laser, propagating along the *z-*axis, is focused to the top surface of the copper blade to produce nanoparticles.

When a multi-GeV stable electron bunch is anticipated based on nanoparticle-based injection, the generated nanoparticles must be localized at a fixed position along the main laser propagation direction. In this article, we propose a unique method to overcome the challenge, localizing the nanoparticle position within 100 μm and thus stabilizing electron injection. The experimental realization of this method was performed at CoReLS with a 5-Hz Ti: Sapphire laser that delivers 1.5 J on target with a pulse duration of 25 fs as main laser pulse. A thin copper blade was inserted above the nozzle and a 532-nm, 3-ns laser pulse ablates the top surface of the copper blade to produce a nanoparticle plume, as shown in Fig. 2. This setup assures that the nanoparticle plume occupies a narrow sheet region orthogonal to the main laser pulse. Thus, the main laser pulse meets the nanoparticles within a small distance below 100 μm during its propagation through the plasma medium along the *x*-axis, which provides a chance to induce a longitudinally localized injection.

The temporal delay between the ablation laser and the main laser pulse is determined by the ablation process for nanoparticle generation and the propagating time from the copper target to the beam path of the main laser. The ablation process of the copper target occurs in several nanoseconds and the expansion velocity ${\text{v}}_{\text{np}}$ of the nanoparticle plume is 10–30 km/s^25^. When the plume arrives at the height of the main pulse, electron injection occurs once a nanoparticle within the plume moves into the focal volume of the main laser pulse. Experimental results indicate that the optimal delay between the ablation laser and the main laser is 0.15–0.35 μs, which matches well the estimated value based on nanoparticles velocities, $\Delta {\text{t}}{=} \Delta {\text{z}}/{\text{v}}_{\text{np}}{=0.17-0.51 \upmu {\rm s}}$.

**Figure 3:**
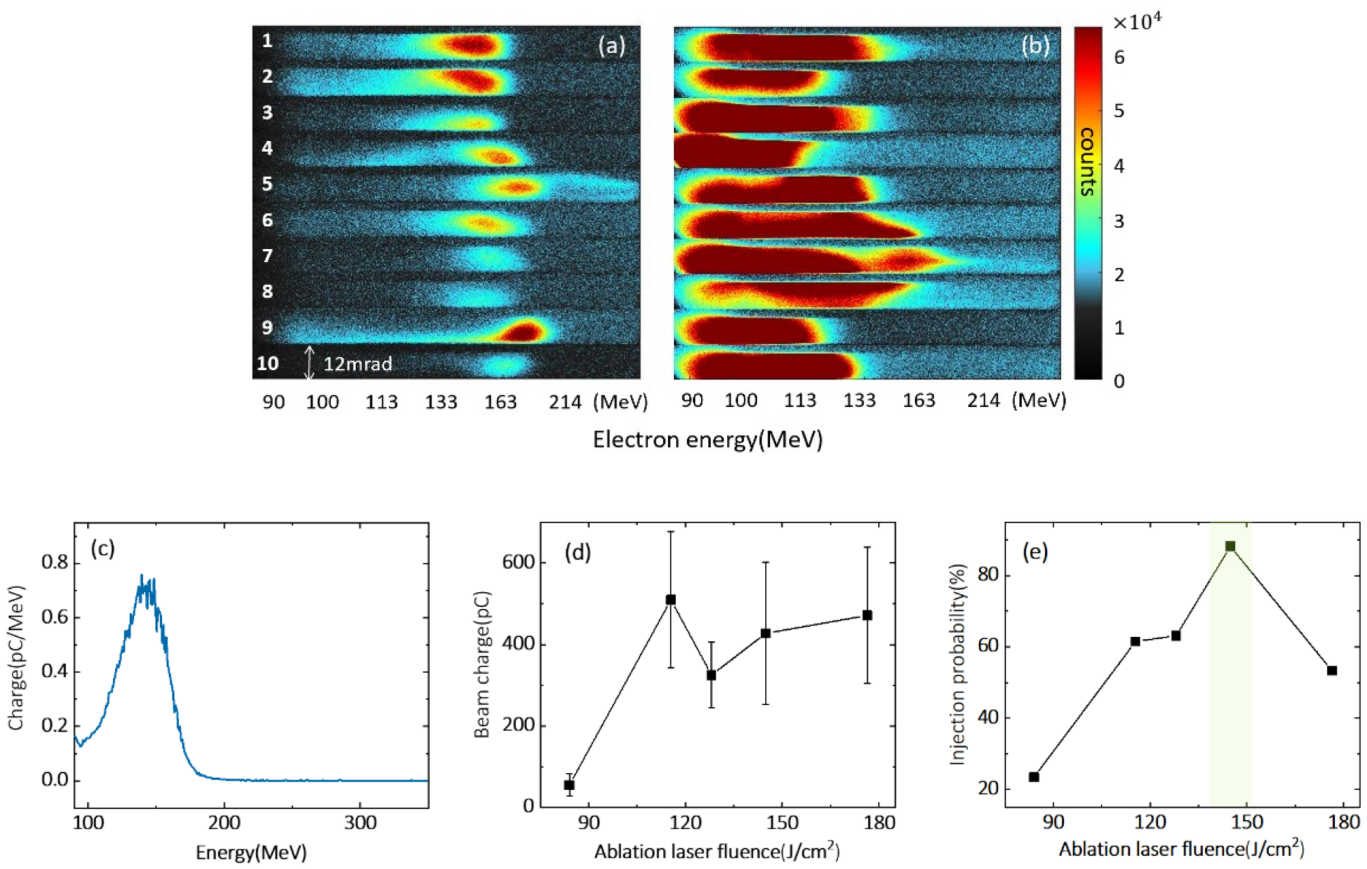
Electron bunch generation with respect to the fluence of the ablation laser. Electron energy spectra of ten laser shots, recorded on Lanex 2, are compared for the ablation energy densities of (**a**) 84 and (**b**) 145 $\mathrm{J}/{\mathrm{cm}}^{2}$. (**c**) A typical energy spectrum of electron bunch with ablation laser energy of 84 $\mathrm{J}/{\mathrm{cm}}^{2}$. Both beam charge (**d**) and injection probability (**e**) were measured while changing the fluence of the ablation laser.

The ablation laser fluence is the main factor that determines the nanoparticle size and density inside the plume. The electron bunch barely appeared as the ablation laser energy was below 5 mJ, where the nanoparticles were produced with a very low density such that the possibility for nanoparticles to meet with the focused laser pulse would be small. When the ablation laser energy increased to 12 mJ, i. e. its fluence of 84 J/cm^2^, the electron bunch signal emerged with a small divergence of 6 mrad as recorded in Lanex 1. Figure 3a displays these electron bunches recorded in Lanex 2 for 10 shots. The electron beams have a narrow energy spread with an average beam charge of 55 pC. Figure 3c plots the lineout of the energy spectrum and shows a single peak at 143 MeV with a relative energy spread of 30%. The energy peak of produced electron bunches stays between 140 and 170 MeV with an average energy of 155 MeV. The observed electron signals in Lanex 1 show that nanoparticles successfully trigger the electron injection. These electron bunches disappeared as the ablation laser was switched off. The stable output of a single energy peak in Lanex 2 implies the advantage of localizing the electron injection process within 100 μm along the main laser propagation axis.

The electron bunches with narrow-energy spread appeared with 23% probability under the ablation laser energy fluence of 84 J/cm^2^ based on data statistics over 200 shots. When the ablation fluence increased to 110 J/cm^2^, the injection probability by nanoparticles increased above 50%, as shown in Fig. 3e. It indicates that the nanoparticle density at the propagation path of the main laser pulse propagation is sufficiently high to support reproducible electron injection. Moreover, the bunch charge was raised to beyond 300 pC with broad energy spectra spreading up to 150 MeV, as plotted in Fig. 3b,d. Here we could not observe the electrons with energy below 90 MeV in Lanex 2 due to the limitation of the magnet exit size, but the beam charge was counted for all the accelerated electrons recorded on Lanex 1. Subsequently, the ablation fluence increased to 145 J/cm^2^, and it is remarkable to find out that the electron bunches were detected with a probability as high as 90% with wide energy spread. One possible reason for the beam charge enhancement is larger nanoparticle size due to the high ablation fluence, which is confirmed by the following nanoparticle characterization in Fig. 5d,e. In virtue of the localized insertion of nanoparticles, the stability and beam charge of *e*-beams can be controlled by adjusting the fluence of the ablation laser so as to choose the density and the size of nanoparticles, while this flexibility in case of non-localized nanoparticle insertion method can diminish due to the uncertainty of the injection position.

**Figure 4:**
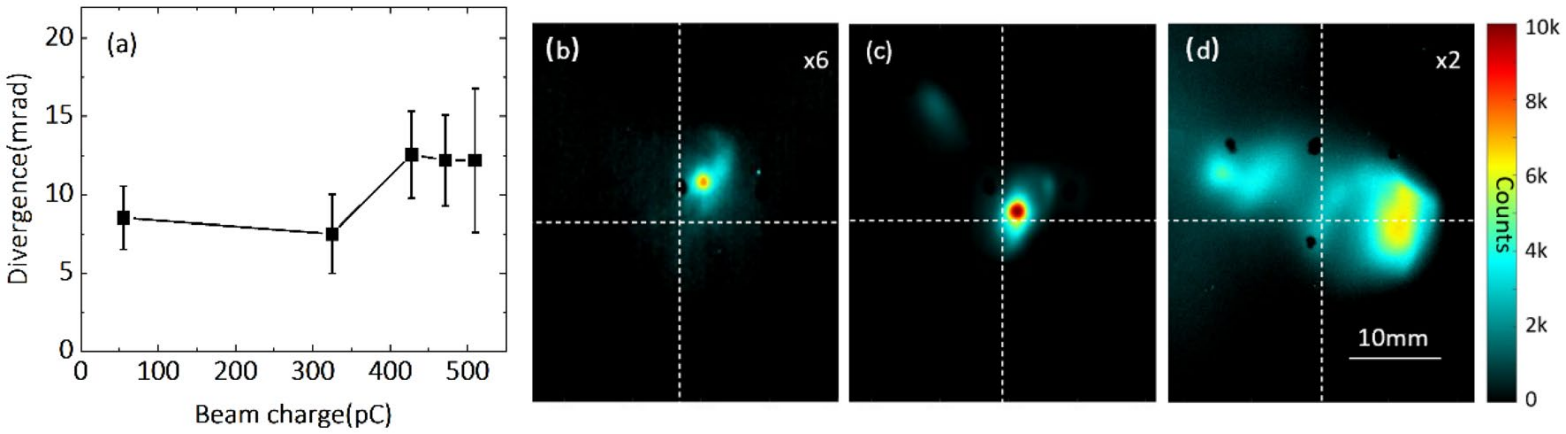
(**a**) Variation of beam divergence with the beam charge of an electron bunch. Here the beam charge of an electron bunch is enhanced as the laser fluence increases from 84 to 176 J/cm^2^. (**b–d**) Raw data of the beam distribution of an electron bunch recorded on Lanex 1. The ablation laser fluence is (**b**) 84, (**c**) 145 and (**d**) 210 J/cm^2^.

We observed, from the Lanex 1 data, that the average e-beam divergence varied only slightly from 8 to 12 mrad, as plotted in Fig. 4a, although the beam charge increased by almost ten times from about 50 pC to 500 pC. There is no significant increment of beam divergence with larger nanoparticle size because the enlarged size of nanoparticle is a few tens of nanometer, which is several-orders smaller than the plasma wavelength. In either case, we observed a single electron bunch on Lanex 1 as shown in Fig. 4b,c, which indicates that mainly one nanoparticle gets involved in the injection process, even for such0 high-charge beams. When the ablation laser fluence further increased beyond 200 J/cm^2^, multiple electron bunches were detected on Lanex 1. At this high ablation laser fluence, the nanoparticle density is so high that more than one nanoparticle is involved in the injection process for a single shot, as plotted in Fig. 4d. The multiple electron bunches accelerated to different propagation directions. Thus, the ablation laser fluence sets up a limit for tuning the ablation laser to manipulate the acceleration outcome. Consequently, manipulating the ablation laser fluence is a critical parameter to obtain high-charge low-beam-divergence electron beams.

**Figure 5:**
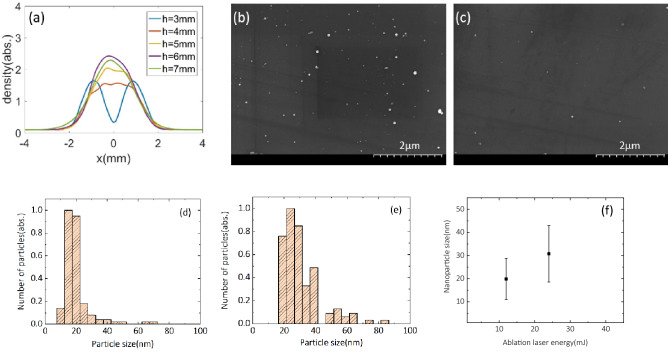
(**a**) Gas density profile at different heights above the nozzle exit calculated from the CFD simulation. (**b**) SEM data of a nanoparticle-deposited silicon wafer surface shows the nanoparticle density reaches maximum at *x* = 0. (**c**) SEM image was taken at *x* = 25 μm. Nanoparticles size distribution with the ablation laser fluence of (**d**) 84 J/cm^2^ and (**e**) 180 J/cm^2^. (**f**) The average sizes and deviations of the nanoparticles with the ablation laser energy.

In order to exclude the effect of gas profile variation by the cooper blade, we simulated the gas profile with the copper blade using the commercial code FLUENT based on a Computational Fluid Dynamics (CFD) modeling^26^. The chosen nozzle and copper blade geometry was the same as the experimental condition. The simulation results show that the gas flow is divided by the copper blade and merged again after flowing 4 mm above the nozzle exit. The longitudinal density profile has two individual density peaks at *h* = 3 mm in Fig. 5a, here *x* = 0 is the center of the gas nozzle. The gas flow starts to combine around 4 mm away from the nozzle exit, and the two density peaks are smeared. The density distribution recovers to an ordinary Gaussian-like profile above *h* = 6 mm. Figure 5a shows that the gas density profile at the height above 5 mm from the nozzle exit becomes a smooth gas profile, which is already sufficient to exclude the effect of gas disturbance by the cooper blade; consequently, we set the main laser pulse propagation path 6 mm away from the nozzle exit when the ablation laser was turned on. The formation of a smooth plasma channel with a length of about 3 mm was observed from the top-view CCD camera in the experiments.

The experimental results demonstrated that the localized nanoparticle insertion is an essential condition that enables the nanoparticle parameters to optimize the electron beam properties for high-charge stable electron beam. To characterize the copper nanoparticles, we inserted a silicon wafer 18 mm above the top surface of the copper target. The nanoparticles were deposited on the silicon wafer, allowing us to learn the spatial distribution and size of nanoparticles. The silicon wafer was exposed by the nanoparticle flume produced only by the ablation laser with an angle of 5º to the copper target. The copper target also moved with 100 μm step size in the *y-*axis after every five shots. Figures 5b,c show the scanning electron microscope (SEM) images of nanoparticles on the silicon wafer. The nanoparticles are close each other within a few μm because the wafer collected nanoparticles for 100 shots. In addition, the nanoparticle density reached maximum at *x* = 0 and dropped significantly as the position shifts by 25 μm either side along the *x*-axis, as shown in Fig. 5c. The nanoparticle distribution on silicon wafer explicitly shows that the nanoparticles occupy a small region of $\Delta x<100 \upmu \mathrm{m}$. In addition, previous numerical study^21^ also showed that the efficient electron injection can occur when the nanoparticle is located a quarter of laser beam diameter. Therefore, one or few nanoparticles could be involved efficient electron injection at this nanoparticle distribution. The designed setup for nanoparticle generation with a thin copper blade has provided a very localized region along the laser propagation axis for electron injection and thus stable energy output, which is consistent with the experimental observation showing the electron energy between 150 and 170 MeV.

The dependence of nanoparticle size on ablation laser fluence can be obtained from the SEM images of nanoparticle-deposited silicon wafers. Figure 5d–f show the nanoparticle size distribution with different ablation laser fluence after the accumulation of 100 shots. For the ablation laser fluence of 84 J/cm^2^, the nanoparticle size is in the range of 10–20 nm, with an average size of 19.9 nm. For the laser fluence of 180 J/cm^2^, the nanoparticle size is distributed between 20 and 40 nm with the average size of 30.8 nm and maximum size up to 90 nm. The electron density of an ionized copper nanoparticle is extremely high and generates an intense local electric field to trigger electron injection. The electric field is linear to the electron charge provided by a nanoparticle, $Q=4\uppi nq{r}^{3}/3,$ where *n*, *q,* and *r* are the atomic density, the charge state of copper ions, and the radius of the nanoparticle, respectively. As large-sized nanoparticles provide much stronger modulation of plasma wakefield, more electrons can be injected into a plasma bubble. We observed the electron bunch charge above 400 pC in experiments at the ablation fluence of 145 J/cm^2^, which is almost ten times higher than that at the ablation laser energy of 84 J/cm^2^. This indicates that the injected charge depends on the size of nanoparticles that can be controlled by the fluence of the ablation laser, as shown in Fig. 5f. Moreover, the modulation of plasma wakefield for a small-size nanoparticle is weak, and the injection with a small nanoparticle can be more sensitive to the fluctuations of laser conditions compared to the case of a large-size nanoparticle. In the experiment, the probability of triggering electron injection was 23% with the ablation laser fluence of 84 J/cm^2^, as shown in Fig. 3a, while the output stability increased above 90% as the ablations laser fluence increased to 145 J/cm^2^. This result showed that the control of ablation laser fluence provided the reliable electron injection from large-size nanoparticles; the electron injection process becomes much less dependent on the self-injection process susceptible to laser fluctuations. Furthermore, nanoparticles with higher material densities can provide higher nanoparticle field with same nanoparticle size, which can offer a chance to generate high-charge electron bunch with quasi-mono-energetic spectrum as expected in previous numerical study^21^. The localized nanoparticle insertion method can, thus, provide a suitable electron injector for staged LWFA, since the small beam divergence can provide an efficient beam loading in the second wakefield structure^27,28^.

## Conclusion

The nanoparticle insertion method to trigger electron injection in LWFA was successfully examined to decouple electron injection from laser evolution. The 3D PIC simulation results showed that it induced negligible effects on laser pulse evolution in a plasma. We have experimentally realized this controllable electron injection method using the laser ablation of a thin copper blade, where the injection length was only about 100 μm along the propagation direction of the main laser pulse. We demonstrated the stable generation of electron bunches with a charge of 100 s’ pC, a broad energy spectrum and small divergence angle by optimizing the laser ablation parameters that determine nanoparticle properties. Consequently, the stable high-charge low-beam-divergence electron beams, initiated by localized nanoparticle insertion, can bring significant advantages of efficient electron injection for a long-distance electron acceleration beyond 10 GeV in LWFA and high flux x-ray generations.

## Methods

### Experimental setup

A 5-Hz Ti: Sapphire laser at CoReLS is employed to study the electron acceleration process. The laser pulse was focused by an *f*/11 spherical mirror into the gas jet with a full-width-half-maximum (FWHM) focal size of 15 μm, corresponding to the peak intensity of 1 × 10^19^ W/cm^2^. A pulsed nozzle served as the gas jet and provided a 3-mm-long helium gas target. A copper blade with a thickness of only 0.1 mm and a height of 0.8 mm was inserted above the nozzle as the ablation target for nanoparticle generation, as shown in Fig. 2. The distance between the blade bottom and the nozzle exit was around 100 μm, which allows the copper blade to move freely in the *x*–*y* plane. A 532-nm, 3-ns laser pulse with an energy density of 80–210 J/cm^2^ and focal size of 65 μm ablated the top surface of the copper blade to produce a nanoparticle plume. The created nanoparticle plume spreads to the normal direction of the blade top surface, i. e. along the *z*-axis, as shown in Fig. 2. In this way, nanoparticles pass through the beam path of the main laser pulse and trigger electron injection when they overlap with the focal volume of the main laser pulse. The copper target moved by 100 μm along the *y*-axis after every 5 shots because the nanoparticle generation and propagation direction strongly depend on the fresh surface of the ablation target^29^.

The main laser pulse in our experiment was set 6 mm away from the nozzle exit to drive a strong nonlinear plasma wave below the self-injection threshold at the gas pressure of 25 bar (gas density of $\text{1.5} \times {10}^{18} \, {\text{cm}}^{-{3}}$). This distance is monitored by a side-view CCD to image the plasma channel. The main laser pulse and the ablation laser pulse were precisely aligned with the help of a top-view CCD to assure their spatial overlap. The electron detection system consisted of one electron spectrometer and two pieces of Lanex screen. The *e*-beam spectrometer with a dipole magnet of *B* = 0.996 T was placed 43 cm away from the gas nozzle. Lanex 1, set in front of the spectrometer, was used to measure the bunch divergence, pointing, and beam charge. A 15 μm-thick aluminum foil blocks the laser beam for Lanex 1. The high-energy electrons are dispersed by the spectrometer and reach Lanex 2 behind the magnet exit. Both Lanexes are imaged by CCD cameras (FLIR Microline and GS3-U3-50S5M-C) with lenses (SAMYANG 2.8/100 mm and 1.4/85 mm). The open area of the magnet exit is $\text{8 mm} \, \times 70\, \text{mm}$, which allows us to measure the electron energy spectrum above 90 MeV with a full divergence angle of 12 mrad^30^.

### Simulations

We have performed 3D PIC simulations by the commercial code VORPAL to study laser pulse evolution process for the nanoparticle-induced electron injection in a low-density plasma^31^. The moving window has the size of 200 × 160 × 160 μm^3^, divided into 2000 × 800 × 800 cells. The laser pulse, linearly polarized along the *y*-axis, propagates along the *x*-axis with a peak intensity of *I*_0_ = 2 × 10^19^ W/cm^2^, pulse duration of 35 fs and FWHM focal size of 70 μm, which comes from the experimental condition for 7.8 GeV electron bunch generation^19^. The background electron density is 3.5 × 10^17^ cm^−3^ and the ionized nanoparticle is set at ${\text{x}} \, {=} \, \text{100 }\, \upmu \text{m}$ with diameter of 100 nm and density of 100*n*_c_, here *n*_c_ is the critical density for the laser center wavelength of 800 nm. Each cell contains 1 macro-particle for background electrons and 10,000 macro-particles for the nanoparticle electrons. Another simulation without nanoparticles was also performed for comparison. Both simulations end at *x* = 300 μm since we are only interested in the laser pulse evolution during the injection process.

## Data Availability

The data that support the findings of this study are available from the corresponding author upon reasonable request. All data generated or analysed during this study are included in this published article.
